# Eggshell coloration and its importance in postmating sexual selection

**DOI:** 10.1002/ece3.2664

**Published:** 2017-01-14

**Authors:** Miroslav Poláček, Matteo Griggio, Ivan Mikšík, Michaela Bartíková, Manfred Eckenfellner, Herbert Hoi

**Affiliations:** ^1^ Institute of ZoologySlovak Academy of SciencesBratislavaSlovakia; ^2^ Department of Integrative Biology and EvolutionKonrad Lorenz Institute of EthologyUniversity of Veterinary Medicine, ViennaViennaAustria; ^3^ Department of BiologyUniversity of PadovaPadovaItaly; ^4^ Department of Analytical ChemistryFaculty of Chemical TechnologyUniversity of PardubicePardubiceCzech Republic; ^5^ Neufang 6FeuersbrunnAustria

**Keywords:** differential allocation, eggshell coloration, female quality, paternal investment, sexual selection, Tree Sparrow

## Abstract

Avian eggshell color seems to fulfill multiple functions, some of them being structural and others signaling. In this study, we tested whether or not eggshell coloration may play a role in sexual selection of Tree Sparrows (*Passer montanus*). According to the “Sexually selected eggshell coloration” hypothesis, eggshell coloration signals female, egg or chick quality and males adjust parental investment according to this signal. Eggs of this species are covered with brown spots and patches, and variation between clutches is high. We found that eggshell coloration correlates with both protoporphyrin and biliverdin, but protoporphyrin concentrations are ten times higher. Eggshell coloration reflects egg and offspring quality, but not female quality. Thus, eggshell coloration may signal female postmating investment in offspring rather than female quality. Furthermore, differential allocation in terms of maternal investment is supported by the fact that females lay more pigmented clutches when mated to males with bigger melanin‐based ornaments relative to their own. Moreover, males invested proportionally more to chicks that hatched from more pigmented clutches. Our correlative results thus seem to support a role of sexual selection in the evolution of eggshell coloration in birds laying brown eggs, pigmented mainly by protoporphyrin.

## 1 Introduction

Many different functions have been proposed to explain the evolution of eggshell coloration (Hanley, Cassey, & Doucet, 2013; Kilner, 2006; Underwood & Sealy, 2002). Avoiding predation was (Wallace, 1889) and still is considered to be the main selective pressure (Kilner, 2006). However, many more hypotheses have been developed to explain egg pigmentation. Some consider egg pigments in relation to a structural function (Bakken, Vanderbilt, Buttemer, & Dawson, 1978; Gosler, Higham, & Reynolds, 2005; Ishikawa et al., 2010; Lahti, 2008), and others as a signal to recognize eggs of intra‐ as well as interspecific brood parasites (Davies & Brooke, 1989), or own eggs in dense breeding situations (Birkhead, 1978). Blackmailing a male via vivid colors to incubate or feed an incubating female in order to keep the eggs concealed (Hanley, Doucet, & Dearborn, 2010) and the unpalatability of eggs (Cott, 1948; Swynnerton, 1916) were also suggested. One recent explanation that considers the potential signaling function of the eggshell color is the “sexually selected egg shell coloration” hypothesis (SSECH; Moreno & Osorno, 2003). The SSECH posits that eggshell coloration is sexually selected and signals female quality to her mate. Based on this signal, males may change their parental investment accordingly (Moreno & Osorno, 2003). In the last decade, this hypothesis has attracted considerable interest, and in this context, major attention has been paid to species with blue‐green eggs, which are based on biliverdin. For this type of pigmentation, there are both correlational evidence and experimental evidence for key predictions of the SSECH (English & Montgomerie, 2011; Fronstin, Doucet, & Christians, 2016; Hargitai, Herényi, & Török, 2008; Morales, Sanz, & Moreno, 2006; Moreno, Lobato, Merino, & Martínez‐de la Puente, 2008; Moreno, Lobato, et al., 2006; Moreno, Morales, et al., 2006; Soler, Navarro, Contreras, Avilés, & Cuervo, 2008, but see Hanley & Doucet, 2009; Honza, Požgayová, Procházka, & Cherry, 2011; Johnsen, Vesterkjær, & Slagsvold, 2011; Krist & Grim, 2007).

Beside biliverdin‐based eggshell color, there is also some evidence for passerine species producing colorful (red‐brown) eggs based on protoporphyrin. The signaling function of protoporphyrin, however, seems less clear. Similar to biliverdin examples, some studies revealed a positive relationship between female quality and protoporphyrin pigmentation (Giordano, Costantini, Pick, & Tschirren, 2015; Holveck et al., 2012; Sanz & García‐Navas, 2009; Stoddard, Fayet, Kilner, & Hinde, 2012; Walters & Getty, 2010). By contrast, female Blue Tits (*Cyanistes caeruleus*), which laid more spotted eggs, were in poorer condition and had higher levels of stress protein and lower levels of immunoglobulins (Martínez‐de la Puente et al., 2007). Similarly, in the House Wren (*Troglodytes aedon*), eggs with less brown pigment were heavier and chicks that hatched from these eggs were fed at higher rates by foster females but not males (Walters & Getty, 2010). Thus, the question arises whether protoporphyrin might in some situations elicit paternal support in line with the compensation hypothesis (Gowaty et al., 2007) rather than indicating female superior quality. As originally proposed, the SSECH predicts a positive association between female or offspring quality and eggshell coloration (Moreno & Osorno, 2003) based on the differential allocation hypothesis (Burley, 1986; Sheldon, 2000).

Costs of eggshell pigments are generally associated with the physiological processes, and these pigments are involved in within the female body. Biliverdin is a strong antioxidant (Kaur et al., 2003; McDonagh, 2001), and protoporphyrin acts as a pro‐oxidant (Afonso, Vanore, & Batlle, 1999). Based on this information, some authors assumed that the two pigments might convey different information or that they signal opposing characteristics (Martínez‐de la Puente et al., 2007; Walters & Getty, 2010). This does not seem to be supported when examining the relationship between eggshell pigment concentrations because in several species protoporphyrin and biliverdin were found to be positively correlated (Cassey, Mikšík, et al., 2012; Cassey, Thomas, et al., 2012; Wang et al., 2009).

**Figure 1:**
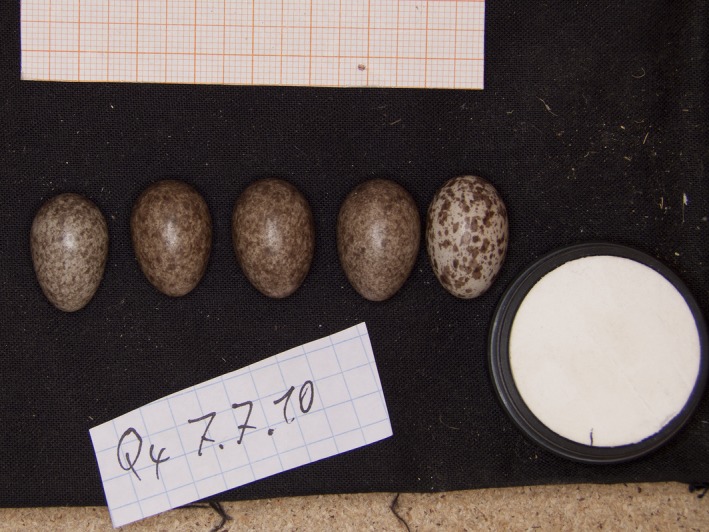
Clutch of tree sparrow, *Passer montanus* (photograph by Miroslav Poláček)

In this study, we used Tree Sparrows (*Passer montanus*) as a model species to investigate the role of postmating sexual selection, in particular the SSECH. They are ideal for such an investigation, because they breed in nest boxes, and we already demonstrated the existence of intensely colored eggs (Figure 1) and a high interclutch variability (Poláček, Griggio, Bartíková, & Hoi, 2013). Nest depredation is low, and interspecific parasitism does not exist; therefore, these two options can be neglected as an important driving force for egg coloration. Tree Sparrows are in particular suitable to investigate the SSEC hypothesis, because male Tree Sparrows invest in chick feeding. Males visit nests regularly during all breeding stages and are potentially able to evaluate clutch characteristics. Moreover, Tree Sparrows have a flexible breeding system ranging from polyandry over monogamy to polygyny. Thus, strategies exploiting male parental care might be essential to increase female reproductive success.

A basic assumption for any signaling function is that eggshell coloration reflects eggshell pigment concentrations (Cassey, Mikšík, et al., 2012). Therefore, we first determined the relationship between eggshell coloration based on pictures processed in Adobe Photoshop using CIE Lab color space and eggshell pigments based on chemical analyses of eggshells. We then tested assumptions derived from the SSECH (Moreno & Osorno, 2003) namely that egg color correlates with (1) female or (2) offspring quality and (3) male feeding investment. Lastly, we wanted to know whether females adjust eggshell coloration according to mate quality and in this way demonstrate maternal investment in a particular brood (Morales, Torres, & Velando, 2012).

## 2 Methods

### 2.1 Study species and fieldwork

Tree Sparrows were studied in an agricultural landscape of Lower Austria, near the village of Feuersbrunn (48°26′N, 15°47′E). Our study population was breeding in nest boxes installed on fence posts in vineyards (288 in 2010, 296 in 2011) and in trees in an apricot orchard (324 in 2010 and 339 in 2011). Pairs usually had three broods per year, with an average of 5.1 ± 0.05 (mean ± *SE*) eggs. Nest building started in the first week of April. Hence, from the beginning of April, all nest boxes were checked weekly. Occupied nest boxes (with nest material) were then checked daily (Poláček et al., 2013).

The quality of the adult birds was estimated using (1) morphological measurements, (2) ornament expression, and (3) health status using blood parameters. To avoid nest desertion, adults were trapped during the nestling stage, either in the nest box or by mist nets close by. After trapping, basic morphological measurements (tarsus length, wing length, and body mass) of adult birds were taken according to Svensson (1992). A condition index was calculated as the residuals from a linear regression with body mass not explained by size (tarsus length). Adult birds were also photographed with a size standard and held in standardized position. Afterward, the area of melanin‐based ornaments (throat patch and cheek patches) was determined using Photoshop. To complete the picture of individual body condition, basic physiological measurements of the blood were taken. Blood was drawn from the brachial vein by 75‐μl‐heparinized hematocrit capillaries and stored at temperature 4°C until processed. Blood was than centrifuged for 4 min at 5,000 rpm to separate plasma from blood cells. Hematocrit was than calculated as a ratio of blood cells to the whole blood.

The quality of nestlings was calculated using body mass at 5 days old (hatching day is day 1). Regarding egg size, we measured egg width and length with a caliper and derived egg volume from these measurements according to Hoyt (1979).

As an estimate of investment, feeding frequency was observed directly by telescope. To ensure natural and undisturbed behavior, birds were observed from a portable hide placed ~10–15 m from the nest box. Alternatively, in some cases a small digital camera, fixed on a tripod, was used. The distance of the camera from the nest box was between 2 and 5 m. Observations were conducted between the days 7 and 9 of nestlings' age in the morning (between 6:00 a.m. and 11:00 a.m.), in two sessions per nest in different days. Total observation time per nest box was between 60 and 120 min. The proportion of male feeding attempts relative to all feeding attempts was then used in the analyses.

### 2.2 Eggshell coloration and pigments

Egg shell coloration data were obtained using digital photography, using Fuji Finepix S200‐EXR. Digital pictures of clutches were taken after the beginning of incubation when the clutch was completed. Clutches were photographed in standardized conditions inside wooden box illuminated by a ring flesh on a black background, and white standard (Top Sensor Systems WS‐2) was always included. Pictures were saved as RAW files and were processed in Adobe Photoshop. CIE Lab color space was used for color measurements. For each egg, four measurements were taken from randomly chosen places evenly distributed on the egg. These four measurements covered together on average 9% of the egg surface area on the picture. The average of these measurements was further used to analyze Lightness (L*), which represents the achromatic properties of an object, ranging from 0 (black) to 100 (white) (for details see Poláček et al., 2013). Later, in this paper, we refer to this variable simply as egg pigmentation.

To support our color measurements with actual amount of pigment in eggshell, we collected unhatched eggs or eggs from abandoned clutches (in total 13 eggs of seven clutches). After collection, these eggs were stored in dark and dry conditions until pigment extraction. Protoporphyrin IX and biliverdin were quantified in the form of their dimethyl esters (Mikšík, Holáň, & Deyl, 1996). Pigments were extracted and esterified in absolute methanol (15 ml) containing concentrated sulfuric acid (5%) at room temperature in the dark under N_2_ for 24 hrs. Extract solutions were decanted, and chloroform (10 ml) and distilled water (10 ml) were added; then, the mix was shaken. The lower (chloroform) phase was collected, and the upper (aqueous) phase again extracted with chloroform (chloroform phases from both extractions were collected). These phases were washed in 10% NaCl (5 ml), followed by distilled water until the wash solution was neutral. Extracts were evaporated till dry and reconstituted in chloroform (1 ml) with an internal standard (5,10,15,20‐tetra(4‐pyridyl)‐21H,23H‐porphine; 0.01 mg/ml). Commercially sourced standards for quantification (protoporphyrin IX and biliverdin) were treated with the same procedure.

Pigments were identified, and their concentration was quantified by reversed‐phase high‐performance chromatography, using a gradient elution between water and acetonitrile with formic acid. Compounds were monitored by UV absorbance, fluorescence, and an ion‐trap mass spectrometer (using multiple reaction monitoring).

### 2.3 Statistical analyses

Because biliverdin and protoporphyrin were correlated (see Section 3), we used two models to test the potential relationship between pigment concentration and egg pigmentation (shell lightness), namely linear mixed‐effect models (LMM) in the R statistical program version 3.0.0, package “lme4” (Bates, Maechler, & Bolker, 2012). Package “lmerTest” (Kuznetsova, Brockhoff, & Christensen, 2013) was used to obtain degrees of freedom and *p*‐values, and effect size (*R* ^2^) was calculated according to Xu (2003). In these models, one particular pigment was used as dependent variable and color measurement as independent variable. Nest was included as a random factor.

For all following analyses, linear models in the R statistical program version 3.0.0 (R Development Core Team, 2012) were used with egg pigmentation determined by Photoshop (see earlier) as the dependent variable. In all of these analyses, we controlled for year and start of laying, which was defined as the day when first egg of particular clutch was laid. Additionally, we calculated variance inflation factors for every initial model to detect potential collinearity. All values were under 2 and therefore considered to be under a conservative threshold (Zuur, Ieno, & Elphick, 2010).

To investigate the effect of female quality on eggshell coloration, two models were fitted. In these two models, the average egg pigmentation was the dependent variable. In one of these models, the independent variables used to describe female quality were female condition, hematocrit, average egg volume, and clutch size. In the second model, we included female ornaments, throat patch, and cheek patch.

To investigate the relationship between nestling quality and eggshell coloration, we used average nestling body mass per nest on day 5 from the first broods of 2010 and 2011. Average chick body mass was used as dependent variables in the model with average egg pigmentation, brood size, and egg volume as independent variables. Brood size and egg volume were included because they might influence chick body mass.

To test for a potential association between eggshell coloration and male parental investment, we fit the model where relative male feeding trips were used as the dependent variable and average egg pigmentation as independent variables. Additional variables that might influence paternal investment decision were included in the model as independent variables, namely average chick body mass at day 5 and brood size.

To estimate relative difference in quality of paired birds, measurements of body size (wing length and tarsus length) and ornaments (surface of throat patch and cheek patch) of females were subtracted from the male measurements. Resulting values were then used in models as independent variables (difference in tarsus, difference in wing difference in throat patch, and difference in cheek patch) with egg pigmentation as dependent variable.

Values reported in the results are for the final models. These were selected by backward stepwise elimination of nonsignificant terms from the initial model. The initial models are shown in tables. All tests were two‐tailed, and *p*‐values smaller than .05 were considered to be statistically significant. Model fit was validated by inspection of residual plots. If it was necessary, data were transformed.

## 3 Results

### 3.1 Relationship between egg pigmentation and pigment concentrations

**Figure 2:**
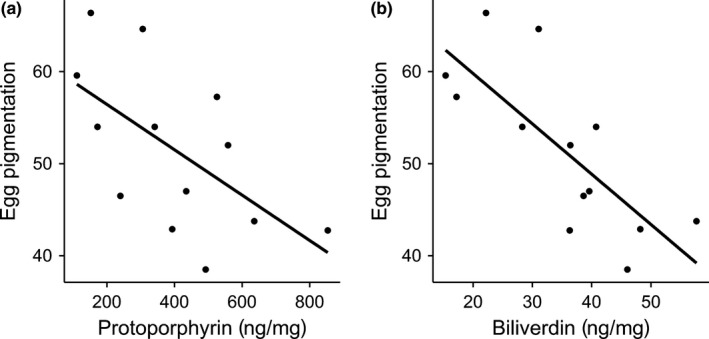
The relationship between average egg pigmentation (ranging from 0—black, to 100—white) per clutch and two eggshell pigments, protoporphyrin (a) and biliverdin (b)

Pigment analyses revealed the presence of both pigments, with protoporphyrin concentration (average concentration ± *SE*: 401.3 ± 59.2 ng/mg eggshell) being more than 10 times higher than biliverdin concentration (average concentration ± *SE*: 35.2 ± 3.4 ng/mg eggshell). Despite the concentration differences of the two pigments, they were significantly positively related (LMM, *F*_1,10.5_ = 10.7, *p* = .007, *R* ^2^ = .94, *n* = 13). Consequently, concentrations of both pigments further correlate with measures we determined for visual egg pigmentation (protoporphyrin: LMM, *F*_1,10.0_ = 6.2, *p* = .03, *R* ^2^ = .40, *n* = 13; biliverdin: LMM, *F*_1,11.0_ = 15.3, *p* = .002, *R* ^2^ = .58, *n* = 13; Figure 2).

**Table 1:** Results of initial linear model testing for the effect of female ornament features on average egg pigmentation based on 16 females

Dependent variable	Predictor	Estimate ± *SE*	*t*	*p*
Egg pigmentation	Throat patch	1.19 ± 1.25	0.952	.362
	Cheek patch	0.377 ± 0.215	1.76	.107
*df* = 11	Start of laying	0.134 ± 0.066	2.04	.066
Adj. *R* ^2^ = .258	Year	−0.93 ± 4.08	−0.228	.824

The variables retained in the final models are indicated by boldface.

**Table 2:** Results of initial linear model testing for the effect of female quality features on average egg pigmentation based on 31 females

Dependent variable	Predictor	Estimate ± *SE*	*t*	*p*
Egg pigmentation	Hematocrit	0.78 ± 1.11	0.703	.489
	**Egg volume**	−**0.024 ± 0.009**	−**2.63**	**.015**
	Condition	−1.66 ± 1.45	−1.15	.261
	Clutch size	−1.89 ± 1.89	−0.998	.328
*df* = 25	Start of laying	0.039 ± 0.05	0.776	.445
Adj. *R* ^2^ = .175	Year	−1.38 ± 3.15	−0.439	.664

The variables retained in the final models are indicated by boldface.

### 3.2 Relationship between egg pigmentation and female, egg, or offspring quality

**Figure 3:**
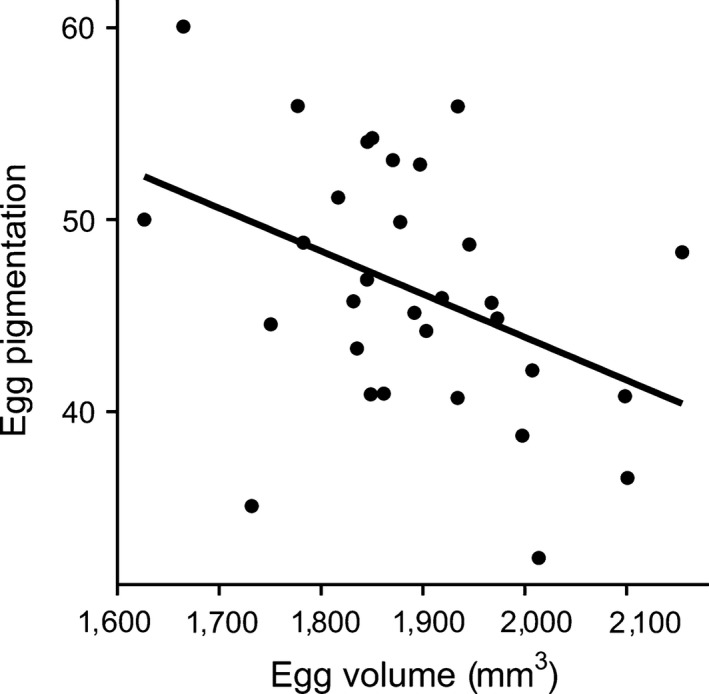
The relationship between average egg pigmentation (ranging from 0—black, to 100—white) per clutch and egg volume

**Figure 4:**
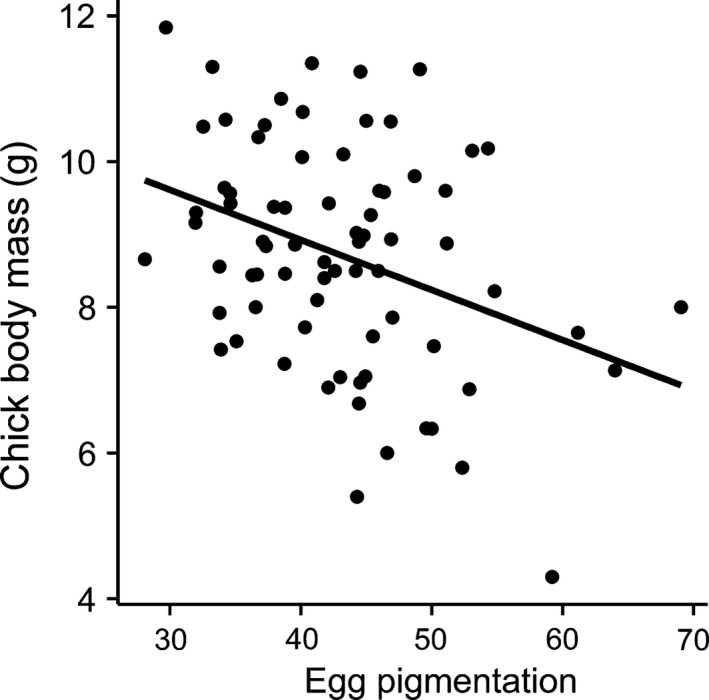
The relationship between the average chick body mass and average egg pigmentation (ranging from 0—black, to 100—white)

There was no relationship between egg pigmentation and the size of female ornaments or condition (Table 1 and 2). However, clutches with more pigmented (darker) eggs had significantly bigger average egg volume (Estimate ± *SE* = −0.022 ± 0.009, *t*_29_ = −2.419, *p* = .022, adj. *R* ^2^ = .14, *n* = 31, Figure 3), and furthermore, chicks that hatched from darker eggs had higher body mass on day 5 (Estimate ± *SE* = −0.069 ± 0.021, *t*_72_ = −3.21, *p* = .02, adj. *R* ^2^ = .11, *n* = 74, Table 3, Figure 4).

**Table 3:** Results of initial linear model testing for the effect of egg features on average nestling body mass per nest based on 74 nests

Dependent variable	Predictor	Estimate ± *SE*	*t*	*p*
Nestling body mass	**Egg pigmentation**	−**0.051 ± 0.026**	−**1.97**	**.054**
	Egg volume	−0.001 ± 0.001	−0.415	.68
	Brood size	−0.084 ± 0.14	−0.601	.55
*df* = 68	Start of laying	0.051 ± 0.04	1.27	.207
Adj. *R* ^2^ = .127	Year	0.702 ± 0.4	1.75	.084

The variables retained in the final models are indicated by boldface.

### 3.3 Egg pigmentation and male feeding investment

**Figure 5:**
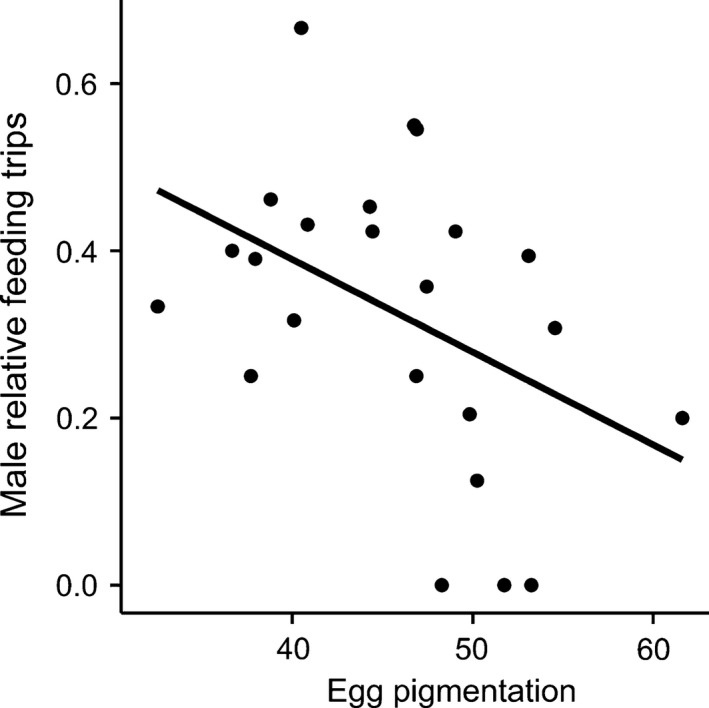
Relationship between male relative feeding trips and average egg pigmentation (ranging from 0—black, to 100—white)

Males fed nestlings proportionally more often in nests with on average darker eggs (Estimate ± *SE* = −0.012 ± 0.005, *t*_21_ = −2.16, *p* = .043, adj. *R* ^2^ = .14, *n* = 23, Figure 5), while other clutch traits were not related to male feeding behavior (Table 4).

### 3.4 Do females adjust eggshell coloration according to mate quality?

Differences in body size between males and females were not significantly related to egg pigmentation. However, we found a significant relationship between egg pigmentation and difference in male and female cheek patch surface. When males had bigger cheek patches comparing to their partner, females laid more pigmented eggs (Estimate ± *SE* = −0.327 ± 0.139, *t*_13_ = −2.35, *p* = .035, adj. *R* ^2^ = .24, *n* = 15, Table 5).

## 4 Discussion

Both pigments are present in Tree Sparrow eggshells, but protoporphyrin concentrations are in an order of magnitude higher than for biliverdin, which is also visually reflected on the shell surface. Tree Sparrow eggs appear mainly brownish, and consequently, one could classify this system as basically protoporphyrin‐based. Despite the different quantities, protoporphyrin and biliverdin concentrations were positively intercorrelated, and thus, darker eggs contained more of both pigments. Such a positive correlation between these pigments was also found by other authors on domestic chicken (Wang et al., 2009) and some wild birds (Cassey, Mikšík, et al., 2012; Cassey, Thomas, et al., 2012) and suggests a similar signaling content of both pigments. Given that protoporphyrin concentrations are more than 10 times higher (see results), the question arises whether a functional or signaling importance of biliverdin is negligible or not? On the other hand, our study does not confirm that the two pigments signal opposing features, an impression one might get when comparing results from studies of species with either biliverdin or protoporphyrin‐based eggs (see Section 1 and Martínez‐de la Puente et al., 2007; Walters & Getty, 2010).

We did not find a relationship between egg pigmentation and female condition or health as formulated in the SSECH hypothesis. We acknowledge that our sample size is quite limited, and this is a correlative study, so we have to be careful when interpreting our results. However, one explanation for the absence of such a relationship could be that female condition changes between egg laying, the time eggshell color was measured, and later during the nestling period, the time females were trapped and measured. On the other hand, such a relationship might not necessarily exist if eggshell coloration signals female investment in a brood rather than her intrinsic quality. As suggested by Morales et al. (2012), males are willing to invest in offspring with a less attractive female when egg color advertises her willingness to invest more in offspring. The underlying assumption in that respect is that egg color reflects egg and offspring quality. This is in fact supported by our results. We found that darker eggs were bigger (Hõrak, Mänd, & Ots, 1997) and egg volume is known to predict nestling quality (Krist, 2011). Egg volume reflects overall maternal investment, including several parameters of maternal investment such as yolk, carotenoids, or yolk testosterone and hence be signaled via eggshell coloration as well. Thus, eggshell pigmentation is not only associated with egg size, but also other components of maternal egg investment that we did not considered in this study. It is for instance known that the intensity of eggshell pigmentation is associated with yolk antibodies (Holveck et al., 2012; Morales et al., 2006) or antioxidant concentration (Hargitai et al., 2008, 2010; Navarro, Pérez‐Contreras, Avilés, McGraw, & Soler, 2011). In support of that, we found chicks hatching from darker eggs were in fact bigger, even when controlling for the effect of egg volume (see Section 3). However, nestling size or condition may depend on not only maternal investment but also posthatching parental investment. In fact, the essential function to explain egg color provided by the SSEC is that females manipulate male offspring investment via eggshell coloration (Moreno & Osorno, 2003). Maternal effects, including eggshell coloration, as a way to manipulate male care are also theoretically supported (Paquet & Smiseth, 2016). Some studies reported a relationship between biliverdin‐based eggshell coloration and male parental investment, either in the form of feeding behavior like in the Pied Flycatcher (*Ficedula hypoleuca*) (Moreno, Morales, et al., 2006; Moreno et al., 2008) and the Spotless Starling (*Sturnus unicolor*; Soler et al., 2008) or incubation effort like in the Blue‐footed Boobies (*Sula nebouxii*; Morales et al., 2012). Similarly, in our protoporphyrin‐based system, we found males to invest proportionally more when broods hatched from more pigmented eggs. Such a relationship for protoporphyrin‐based eggshell coloration was otherwise only found for Blue Tits (Sanz & García‐Navas, 2009). Thus, faster developing (bigger) chicks could be a result of maternal egg investment as well as paternal feeding investment.

According to the “differential allocation” hypothesis, females should adjust their investment into offspring in relation to the attractiveness of their partner (Burley, 1986; Sheldon, 2000). In line with this, it has been already shown that female maternal investment depends on male attractiveness (Gil, Graves, Hazon, & Wells, 1999; Horváthová, Nakagawa, & Uller, 2012; Krištofík et al., 2014; Williamson, Surai, & Graves, 2006) and eggshell coloration on maternal investment for both protoporphyrin‐based eggshell coloration (Holveck et al., 2012) and biliverdin‐based (Morales et al., 2006; Navarro et al., 2011) eggshell coloration. In support of that, we found that in pairs where the male showed comparatively bigger melanin‐based ornaments, the female laid darker eggs and melanin‐based ornaments are in fact known to signal quality, for example, social status and/or body condition (Hoi & Griggio, 2008; Nakagawa, Ockendon, Gillespie, Hatchwell, & Burke, 2007). Because in our study it seems that eggshell coloration does not directly reflect female intrinsic quality, its primary function could be to signal female propensity to invest into offspring and consequently to signal offspring quality to the male (Morales et al., 2012) and this investment may also depend on male attractiveness or quality. Clearly, experimental tests on our and other species are needed to enhance the general reliability of our results. Because of the correlative nature of our study, there are potential alternative explanations for observed relationship between male ornaments and eggshell coloration. One alternative explanation could be that both measured traits depend on local food availability or other environmental conditions. This is improbable because ornaments are formed during autumn molting, while eggshell coloration is formed in the spring, and thus, these events are approximately half a year apart (Summers‐Smith, 1995). Another alternative could be that individuals of similar quality pair together in a process of mutual mate choice. We cannot rule this explanation out completely, but it seems that in our population there was no assortative mating for melanin‐based ornaments (throat patch: Spearman's rank correlation, *r*_s_ = −.104, *p* = .655, *n* = 21; cheek patch: Spearman's rank correlation, *r*_s_ = .089, *p* = .717, *n* = 19). Therefore, experimental studies are required to support this hypothesis.

**Table 4:** Results of initial linear model testing for the effect of egg features on relative male feeding trips based on 23 nests

Dependent variable	Predictor	Estimate ± *SE*	*t*	*p*
Male feeding trips	**Egg pigmentation**	−**0.01 ± 0.006**	−**1.57**	**.134**
	Brood size	0.013 ± 0.035	0.364	.72
	Nestling body mass	−0.017 ± 0.028	−0.604	.554
*df* = 17	Start of laying	−0.002 ± 0.002	−0.995	.334
Adj. *R* ^2^ = .028	Year	0.005 ± 0.103	0.048	.963

The variables retained in the final models are indicated by boldface.

**Table 5:** Results of initial linear model testing for the effect of differences in ornament and body measurements size between paired male and female on average egg pigmentation based on 15 nests

Dependent variable	Predictor	Estimate ± *SE*	*t*	*p*
Egg pigmentation	Difference in tarsus	2.48 ± 3.03	0.819	.436
	Difference in wing	−0.811 ± 1.02	−0.791	.452
	Difference in throat patch	0.046 ± 0.038	1.23	.255
	**Difference in cheek patch**	−**0.257 ± 0.157**	−**1.64**	**.139**
*df* = 8	Start of laying	−0.038 ± 0.085	−0.453	.663
Adj. *R* ^2^ = .36	Year	−5.31 ± 3.93	−1.35	.214

The variables retained in the final models are indicated by boldface.

Contrary to our results, Collared Flycatchers (*Ficedula albicollis*) females laid more pigmented eggs when mated to low‐quality males apparently to compensate for their inferior quality, because more pigmented eggs in this study contained more carotenoids (Hargitai et al., 2008). However, in this case, eggshell coloration also signaled investment in egg quality regardless of reproductive decisions for reproductive compensation (Gowaty et al., 2007) or differential allocation (Burley, 1986; Sheldon, 2000).

As previously noted, some alternative hypotheses can be ruled out by the nature of our model species. Egg predation (Wallace, 1889) is very low in our nest box population; consequently, blackmailing males (Hanley et al., 2010) is also an unlikely function for our Tree Sparrows. Furthermore, interspecific brood parasitism does not exist and intraspecific brood parasitism seems to be rather low (Poláček et al., 2013). A thermoregulatory function (Bakken et al., 1978; Lahti & Ardia, 2016) seems also unlikely given that eggs, well hidden in nest boxes, should be less susceptible to solar radiation or thermal variation.

In conclusion, eggshell coloration seems to reflect aspects of egg and chick quality in tree sparrow. Moreover, males invested proportionally more in chicks that hatched from more pigmented clutches. We found indication that eggshell coloration signals female postmating offspring investment, as females paired to males with relatively bigger melanin‐based ornaments laid more pigmented clutches. Our correlative results thus seem to support a role of sexual selection in the evolution of eggshell coloration in birds laying brown eggs, pigmented mainly by protoporphyrin. An experimental study manipulating ornaments and eggshell colorations of parents may be useful to investigate how these factors affect parental investment.

## Ethics Statement

This research was conducted in compliance with the Guidelines of the Austrian Law. Permission to do this study with the Tree Sparrows in the study area was provided by the Amt der Niederösterreichischen Landesregierung under the licence number: RU5‐BE‐7/010‐2011 and the Ministry of Sciences under the licence number: BMWF‐68.205/0245‐II/3b/2012.

## Conflict of Interest

None declared.
